# The contribution of nitrogen deposition to the eutrophication signal in understorey plant communities of European forests

**DOI:** 10.1002/ece3.2485

**Published:** 2016-12-18

**Authors:** Han F. van Dobben, Wim de Vries

**Affiliations:** ^1^ Wageningen University and ResearchWageningenThe Netherlands

**Keywords:** atmospheric deposition, forest vegetation, plant community dynamics, plant species diversity, resampling study, soil chemistry

## Abstract

We evaluated effects of atmospheric deposition of nitrogen on the composition of forest understorey vegetation both in space and time, using repeated data from the European wide monitoring program ICP‐Forests, which focuses on normally managed forest. Our aim was to assess whether both spatial and temporal effects of deposition can be detected by a multiple regression approach using data from managed forests over a relatively short time interval, in which changes in the tree layer are limited. To characterize the vegetation, we used indicators derived from cover percentages per species using multivariate statistics and indicators derived from the presence/absence, that is, species numbers and Ellenberg's indicator values. As explanatory variables, we used climate, altitude, tree species, stand age, and soil chemistry, besides deposition of nitrate, ammonia and sulfate. We analyzed the effects of abiotic conditions at a single point in time by canonical correspondence analysis and multiple regression. The relation between the change in vegetation and abiotic conditions was analyzed using redundancy analysis and multiple regression, for a subset of the plots that had both abiotic data and enough species to compute a mean Ellenberg *N* value per plot using a minimum of three species. Results showed that the spatial variation in the vegetation is mainly due to “traditional” factors such as soil type and climate, but a statistically significant part of the variation could be ascribed to atmospheric deposition of nitrate. The change in the vegetation over the past c. 10 years was also significantly correlated to nitrate deposition. Although the effect of deposition on the individual species could not be clearly defined, the effect on the vegetation as a whole was a shift toward nitrophytic species as witnessed by an increase in mean Ellenberg's indicator value.

## 1 Introduction

Ecological effects of atmospheric deposition were first noticed in the 1960s (Odén, 1967) and generated extensive public debate, especially after large‐scale forest dieback had been predicted in the 1970s (Ulrich, Mayer, & Khanna, 1979). Today, the focus has shifted from deposition of acidity to deposition of nitrogen compounds, and effects are now defined in terms of biodiversity loss rather than in terms of forest dieback, but there is still great concern about the ecological effects of deposition. Available data suggest that increasing N availability causes an overall increase in plant biomass production accompanied by a shift toward species adapted to a high N availability and usually by an overall decline in species diversity (Aerts & Berendse, 1988; Bobbink et al., 2010; Tilman, 1987). Effects of N deposition are now recognized in nearly all oligotrophic natural ecosystems, at least those in boreal, temperate and Mediterranean climates, and include grassland, heathland, coastal habitats, oligotrophic wetland (mire, bog, and fen), forests, and aquatic and marine habitats (Achermann & Bobbink, 2003; Bobbink, Ashmore, Braun, Flückiger, & van den Wyngaert, 2003; Bobbink & Hettelingh, 2011; Dise et al., 2011; Gilliam, 2006; Pardo et al., 2011). A meta‐analysis of N addition experiments by De Schrijver et al. (2011) showed a significant loss of plant species in grassland and heathland in response to N enrichment. However, in forests, the species loss was not significant. This is surprising as earlier N addition experiments in forest (Falkengren‐Grerup, 1998; Kellner & Redbo‐Torstensson, 1995; Skrindo & Økland, 2002; Strengbom, Nordin, Näsholm, & Ericson, 2001; Van Dobben, ter Braak, & Dirkse, 1999) as well as observational studies in N deposition gradients (Falkengren‐Grerup, 1995a; Seidling, Fischer, & Granke, 2008; Van Dobben & De Vries, 2010) showed significant effects of N deposition on understorey species composition (see Gilliam, 2006 for a review). Moreover, deposition in forest usually exceeds deposition in nearby grassland or heathland by at least a factor two due to a greater canopy roughness (Erisman & Draaijers, 2003).

Both N addition experiments and observational studies along N deposition gradients have their shortcomings. Addition experiments need to be carried out over an extended period to show long‐term effects, using realistic N loads in low background N deposition areas to avoid that major effects have already occurred (De Vries et al., 2010). Gradient studies are necessarily conducted over a large geographical extent and hence deposition may be confounded with other (e.g., edaphic, climatic) factors.

The problem of confounding factors in gradient studies can to a certain extent be overcome by resampling, that is, making repeated observations over a period of increasing deposition (Verheyen et al., 2012). However, when resampling is performed over a prolonged period of, for example, several decades, the problem of confounding pops up again. It cannot be expected that forest management is constant over such a long period, whereas in unmanaged reserves succession in the tree layer will alter the environmental conditions for the understorey vegetation.

In their detailed resampling study of understorey vegetation in forest reserves in Central Europe, Verheyen et al. (2012) found a significant overall increase in Ellenberg's (1991) nutrient availability indicator (*N*), a marginally significant decrease in Ellenberg's light indicator (*L*) but no significant change in species number. However, these changes could be sufficiently explained from succession in the tree layer causing a reduction in light transmissivity and more easily decomposable litter. Mean deposition of total nitrogen over the observation period did not significantly contribute to the explanation of these changes. The results of this study therefore seem to confirm De Schrijver et al.'s (2011) finding that N deposition effects in forest are limited compared to low vegetation. It could however be hypothesized that due to the long observation period of Verheyen et al.'s (2012) study (17–67 years) in combination with the exclusive use of data from forest reserves, the changes in the tree layer have been so large that they mask the effect of deposition on understorey vegetation.

Bernhardt‐Römermann et al. (2015), in a meta‐analysis of 39 resampling studies in European temperate forests with a time interval of 17–75 years between consecutive surveys, concluded that there was an effect of the N deposition, which was however related to the accumulated N deposition before the first observation, rather than the actual N deposition during the observation period or its change. Also, these authors conclude that N‐mediated changes may be delayed if light availability at the forest floor is low, and only become apparent if the total cover of the tree, shrub, or herb layer decreases.

The studies of Verheyen et al. (2012) and Bernhardt‐Römermann et al. (2015) both concentrate on generalized measures [i.e., diversity measures like the number of species or the Shannon‐Weaver index or Ellenberg's (1991) indicator values]. In general, studies on plant diversity versus N deposition tend to concentrate on indices rather than on the species themselves (e.g., Maskell, Smart, Bullock, Thompson, & Stevens, 2010; Stevens, Dise, Mountford, & Gowing, 2004; Stevens et al., 2010). An exception is Payne, Dise, Stevens, Gowing, and Partners (2013) who estimated the impact of N deposition at the species level using a large data set of European grasslands along a gradient of N deposition. Here, we analyze a set of resampling data of the species composition of the ground vegetation in European forests, collected over a short observation period (7–11 years) in normally managed production forest instead of forest reserves. We relate these data to influencing drivers, including N deposition. We do not only use composite indices, but we also pay attention to species assemblages, and their relation with N deposition. The focus on the ground vegetation is warranted because most of the species diversity of forests occurs in this herbaceous layer (Gilliam, 2006). Our data originate from the “International Co‐operative Programme on the Assessment and Monitoring of Air Pollution Effects on Forests” (ICP‐Forests), which was established by the United Nations Economic Commission of Europe (UN‐ECE) under the Convention on Long‐range Transboundary Air Pollution (LRTAP; EC & UN/‐ECE, 2000). In the framework of this program, a set of “intensive monitoring plots” was installed throughout Europe from 1996 onward (the so‐called Level II plots, De Vries et al., 2003). Monitoring at these plots includes the assessment of tree growth, crown condition, chemical composition of foliage and soil, and species composition of the ground vegetation on most plots, whereas atmospheric deposition, meteorological variables and soil solution chemistry are monitored on a subset of the plots. In principle, both biotic and abiotic observations on these plots are repeated at regular intervals, the length of which depends on the variable and may also vary per country. Up to 2006, one or more observations were available for 934 plots divided over 31 countries. Besides the abiotic data measured on each plot, we also used generic climate data and simulated deposition data from the EMEP model (Simpson et al., 2012; Tarrasón et al., 2007) as explanatory variables. This data set allows an analysis of the temporal change in the vegetation and its abiotic drivers on a European scale over a relatively short period, thus avoiding large changes in the tree layer.

The principal aim of our study was to determine the magnitude and direction of the change in the understorey vegetation in managed forests over an observation period of max. 11 (average 9.3) years on a European scale, and to assess the most probable causes for this change. To arrive at a better understanding of the response of forest vegetation to its abiotic environment, we fist analyzed the relation between vegetation at a single point in time on the one hand and abiotic and biotic conditions (i.e., soil, climate, deposition, and dominant tree species) on the other.

## 2 Materials and Methods

### 2.1 Vegetation data

The present analysis is based on the ICP‐Forests data collected up to 2006. Over that period, the database contains vegetation data from 776 plots, divided over 28 countries (see Appendix S1). The vegetation data consist of relevés, which are lists of estimated ground cover percentages per species. From each plot, between one and eight of such relevés made at different points in time are available. Appendix S2 gives the numbers of relevés per plot. Slightly more than half of the plots have been visited more than once, at intervals between one and eleven years (Appendix S3). For the analysis of the temporal changes, we used the first and the last relevé of the plots that have a time series of at least 7 years.

To characterize the vegetation, we used (1) indicators derived from species abundances using multivariate statistics and (2) indicators derived from species presence/absence, that is, means of Ellenberg's (1991) indicator values, and species numbers. For each relevé, Ellenberg indicator “scores” were computed as the unweighted mean over all species of the indicator values for light (*L*), temperature (*T*), continentality (*K*), humidity (*F*), acidity (*R*), and nutrients (*N*), using a minimum of three species with a known Ellenberg value (see Appendix S4). Moreover, following Verheyen et al. (2012), we used Lennon, Koleff, Greenwood, and Gaston's (2001) dissimilarity index to characterize temporal species turnover. Cover percentages per plant species were acquired according to Canullo et al. (2011); a summary of field methods is given by Van Dobben and De Vries (2010). Details on the treatment of the species data are given in Appendix S4.

The analysis of the change over time was based on a comparison of the first and the last relevé of each plot for a subset of plots where the time interval between these relevés was at least 7 years. The time interval itself was not considered in the analysis. For the analysis at a single point in time, the last relevé of each plot was used (incl. those with only one observation or observations at short time intervals). Relevés taken in fenced plots were not used to avoid spurious effects as a result of the exclusion of grazing (Fuller & Gill, 2001; Kuiters & Slim, 2002). The tree layer was not considered as a part of the spontaneous vegetation and not used in the analysis except as an explanatory variable. The moss and lichen layer was left out of consideration because it was not recorded by all countries.

**Figure 1:**
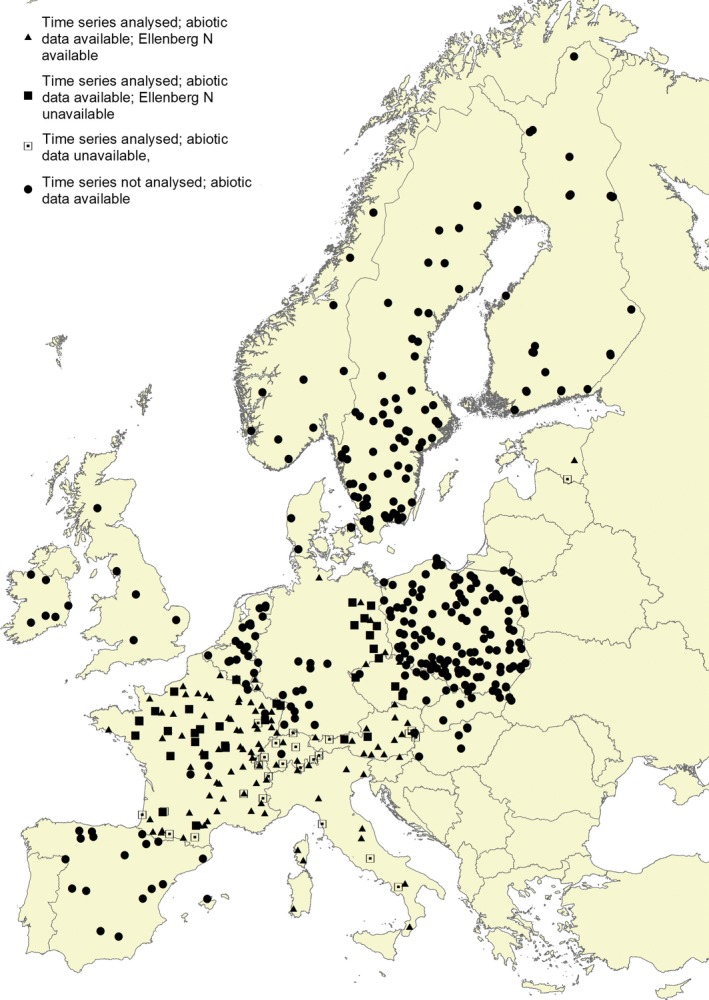
Location of the plots. Explanation of symbols: closed = sufficient abiotic data available, open = insufficient abiotic data; circle = time interval between first and last relevé <7 years, square = time interval >6 years but insufficient species to compute Ellenberg *N* in both years, triangle = time interval >6 years and sufficient species to compute Ellenberg *N*

Figure 1 shows the geographical distribution of the plots and indicates (1) whether they have repeated observations or not, (2) whether they have sufficient abiotic data or not, and (3) whether they have enough species to compute an Ellenberg *N* score. The triangles and squares are the location of 161 plots that have at least two relevés made at an interval of at least 7 years, of which 134 have abiotic data (closed symbols), and 99 have both abiotic data and enough species to compute an Ellenberg *N* score (triangles). The latter subset was used to analyze the relationships of the vegetation changes with abiotic data (see Section Analysis of the relation between vegetation changes and environment). It further shows the location of another 343 plots (circles) with only one relevé and abiotic data. These plots, together with the above 134 plots (thus in total 477 plots), were used to analyze the relation between vegetation and abiotic conditions at a single point in time (see Section Analysis of the relation between vegetation and environment at a single point in time). No analysis was carried out for the remaining 272 (i.e., 776–161–343) plots that did not have abiotic data and time series of less than 7 years.

### 2.2 Soil data

The database contains a wide range of chemical data from a wide range of horizons (De Vries et al., 2000), but the variables included in this study were limited to the generally available ones, that is:

—in the complete organic layer: pH (CaCl_2_) and total contents of Ca, K, Mg, P, C, and N

—in the mineral layer averaged over 0–20 cm depth: pH (CaCl_2_), CEC, base saturation, C‐total and N‐total.

Details on soil sampling and analysis are given by De Vries et al. (2000), and details on data manipulation are given in Appendix S5.

### 2.3 Deposition data

In the ICP‐Forests program, deposition is estimated on a subset of slightly less than half of the plots by chemical analysis of rainwater collected in open funnels both inside the forest (throughfall) and in adjacent open field (bulk deposition). In principle, such data can be used to estimate net deposition onto the forest by applying a canopy budget model that assumes a conservative behavior of Na (De Vries et al., 2001). We did, however, not use these data because of the limited number of plots for which rainwater analyses (both bulk deposition and throughfall) are available. Instead, we used the Eulerian atmospheric transport and deposition model of EMEP/MSC‐W (Tarrasón et al., 2007), which produces estimates of yearly mean deposition of NH_4_, NO_3,_ and SO_4_ on a 50 × 50 km grid; we used values for 1995 and 2000. Van Dobben and De Vries (2010) showed that the predictive power for vegetation of the EMEP‐generated deposition is at least as good as that of the deposition inferred from rainwater analyses. Appendix S6 gives a comparison of the estimates derived by both methods; correlations coefficients appear to be in the order *R* ≈ .6 for both N and S.

### 2.4 Additional data

Each plot was assigned to one of seven climatic zones (boreal; boreal temperate; atlantic north; atlantic south; subatlantic; continental; mediterranean) on the basis of its geographical position, according to De Vries et al. (2002). Moreover, the following data were taken from the ICP database: country; latitude and longitude; stand age; altitude; tree species. Tree species were clustered as follows: for *Quercus*, the species were taken together in two groups: temperate and mediterranean. *Pinus sylvestris* and *Pinus nigra* were taken together. *Fagus sylvatica* and *Picea excelsa* were used as such. All other species were lumped to “coniferous” and “deciduous.” A complete list of tree species is given in Appendix S7.

### 2.5 Analysis of the relation between vegetation and environment at a single point in time

The statistical methods used to relate vegetation characteristics (species abundances, Ellenberg's indicator values and species numbers) to environmental variables at a single point in time are similar to those used by Van Dobben and De Vries (2010). The effect of the environmental variables on the abundances of all species, using the last relevé of each plot, was assessed by canonical correspondence analysis (CCA). Species with less than three occurrences and relevés with less than three species were excluded from the multivariate analysis, to avoid a very heterogeneous data set which hampers the CCA algorithm. Cover percentages were log(*X* + 1)‐transformed. The explanatory variables included were tree species, climate zones, altitude, geographical coordinates, stand age, soil chemical variables, and deposition estimates of NO_3_, NH_4,_ and N‐total. After the exclusion of records with deficient abiotic data (see Appendices S4 and S5), 477 plots remained with both vegetation and abiotic data, with a total of 170 species. Multivariate response models were derived by stepwise addition of explanatory variables. First, a correlation analysis of the explanatory variables was carried out (Appendix S8). Next, in each step, the variable was selected from the pool in Appendix S8 that led to the highest increase in explained variance and was added to the model with the constraint that variables with a correlation |*R*| > .5 with any variable already in the model were skipped, until none remained that could significantly (*p* < .05) improve the fit of the model. In addition to the CCA analysis, the relation between the environmental variables and both the number of species per plot and Ellenberg's (1991) indicator values were assessed by multiple regression.

### 2.6 Analysis of the relation between vegetation changes and environment

There were 161 plots with at least two relevés, which were made with an interval of at least 7 years, containing a total of 297 species. For these plots, the change in the vegetation was determined as (%cover in last relevé) minus (%cover in first relevé) per species. The significance of the change per species was determined by means of a paired *t*‐test. The relation between the change in abundances of all species together and the environmental variables was analyzed using RDA (redundancy analysis, i.e., the linear form of CCA or the canonical form of PCA), and the forward selection procedure explained above, for a subset of 99 of the above 161 plots that had both abiotic data and enough species to compute an Ellenberg *N* score (see also Appendix S4). The significance of the change in Ellenberg scores and number of species per plot was determined by a paired *t*‐test. The significance of the dissimilarity between the first and the last relevé per plot was determined using the Lennon dissimilarity index (Lennon et al., 2001). After the RDA analysis, the composite indicators for change (i.e., change in Ellenberg scores, change in number of species and Lennon dissimilarity index) were projected into the RDA plots as “passive” variables (i.e., showing their correlation with the ordination axes without affecting the ordination itself) to find possible causes for their change.

The temporal change in the composite indicators was related to the environmental variables by multiple regression and backward selection, that is, stepwise removal of nonsignificant terms from a full model containing all environmental variables summed up in Appendix S8, until only variables with a significant effect remained (*p* < .05, based on *t*‐values of regression coefficients).

All multivariate operations were carried out by the program CANOCO v 4.53 (Ter Braak & Smilauer, 2002) and all univariate operations by the program GENSTAT v 13.1 (Payne et al., 2011).

### 2.7 Assessment of the country effects

Before undertaking a regression analysis, possible observer effects were assessed by determining the unique contributions of the countries and the “real” environmental variables to the variance explained by a CCA model containing terms for the countries plus all variables summed up in Appendix S8. Details are given in Appendix S9, which shows that of a total of 25% variance that can be explained anyway, 5% is uniquely due to the countries. As both the geographical coordinates and climate zones were among the “real” environmental variables, this “county effect” is most probably caused by methodological differences. Estonia, France, Italy, Ireland, and the Netherlands are the most deviant countries (in that order), and their effect is significant (*p* < .05) even after accounting for the effect of all “real” environmental variables. Therefore, the countries were used as covariables in the CCA analysis, and the countries were included as an explanatory variable in the univariate analyses. Although the absolute abundance per species (or their probability to be observed) is apparently country‐dependant, it was assumed that their temporal change is not, and therefore, the countries were not included in the analysis of the temporal change.

## 3 Results

### 3.1 Relation between vegetation and environment at a single point in time

#### 3.1.1 Multivariate analysis of impacts of environmental variables on species abundances

Table 1A shows the effect of the environmental variables on species abundances determined by forward selection in CCA. The pH of the organic layer appears to be the most important explanatory variable, followed by tree species and other soil chemical variables, and there is a small, but highly significant effect of NO_3_ deposition. A summary of the model where the variables are taken together per compartment is given in Table 1B. The results strongly agree with those of Van Dobben and De Vries (2010) with the traditional factors (in the order: tree layer, soil, climate) as the most important explanatory variables, and c. 5% of the variance in the fitted values explained by deposition.

**Table 1:** Multivariate regression model explaining the effect of environmental variables on the species abundances of the last relevé of each plot using forward selection in CCA, using the countries as covariables

A				
Variable	Compartment	*F*	*p*	Percentage explained variance
pH	Organic	8.10	.001	1.7
Mediterr. oak	Tree	6.79	.001	1.4
Temperate oak	Tree	5.95	.001	1.3
*Pinus sylv* + *nigra*	Tree	4.83	.001	1.0
Fagus	Tree	4.42	.001	0.9
CEC	Mineral	2.84	.001	0.6
N/C	Organic	2.46	.005	0.5
Latitude	Climate	2.44	.001	0.4
NO3 (2000)	Deposition	2.36	.001	0.5
Longitude	Climate	2.28	.001	0.5
Coniferous “other”	Tree	2.23	.003	0.4
Deciduous “other”	Tree	2.13	.061	0.4
Ca	Organic	2.10	.002	0.4
Atlantic South	Climate	1.96	.008	0.4
Age	Tree	1.89	.003	0.3
Atlantic North	Climate	1.85	.005	0.3
K	Organic	1.87	.011	0.4
Boreal	Climate	1.72	.012	0.3
P	Organic	1.70	.007	0.3
Altitude	Climate	1.36	.092	0.3
N/C	Mineral	1.23	.173	0.3
Further terms not given				
Sum if *p* < .05				12.4

B		
Compartment	% Explained variance (data)	% Explained variance (fitted values)
Tree layer	5.9	47.3
Soil organic layer	3.4	27.4
Soil mineral layer	0.6	4.8
Climate	2.0	16.4
Deposition	0.5	4.1
Sum	12.4	100.0

Eigenvalues: λ_1_ = 0.257, λ_2_ = 0.235, λ_3_ = 0.185, λ_4_ = 0.120, sum of all eigenvalues = 11.739, sum of all canonical eigenvalues = 1.458, number of plots = 477, number of species = 170. Rare species are downweighted. *F* = (regression mean square with this term—regression mean square without this term)/error mean square; *p* = probability of this, or a higher *F*‐value under the null hypothesis as determined on the basis of 999 bootstrap samples. The pool of environmental variables from which the terms were selected included all soil chemical variables, tree species, and climate zones; and altitude, geographical coordinates, stand age; and EMEP deposition estimates of NO_3_ and NH_4_ for both 1995 and 2000, with the constraint that their mutual absolute correlation coefficient should always be below 0.5 (see Appendix S8). A: the selection results per variable, B: a summary per compartment giving the percentage explained variance with respect to the data (left column) and with respect to the fitted values (right column).

**Figure 2:**
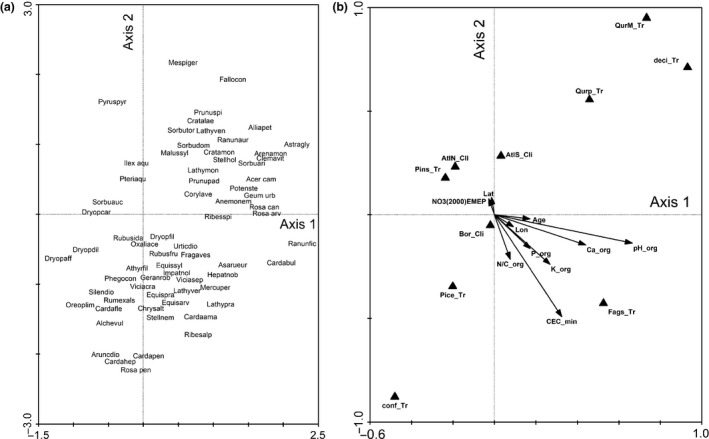
Biplot of the canonical correspondence analysis (CCA) model in Table 1. (a) First and second axis, species; (b) first and second axis, environmental variables. See Table 1 for details of the model, percentage variance in the fitted values explained by this figure: 34% (i.e., (λ_1_ + λ_2_)/Σλ_can_). The plotted species are a selection of species with the highest percentage variance explained by the model, excluding tree saplings. To form a biplot, the plots A and B have to be projected over each other in equal scaling. Quantitative variables are indicated by arrows, class variables by triangles. Projecting the center of a species’ name on an arrow for a quantitative variable gives an approximation of the fitted value of the species’ optimum with respect to that variable, with scaling: origin = mean, head of the arrow = mean plus one standard deviation, mirror image of head with respect to origin = mean minus one standard deviation. Species whose names coincide with a triangle representing a class variable have their optimum in that class. Explanation of environmental variables: Lat, Lon: geographical latitude, longitude, Age: stand age, NO3(2000)EMEP: deposition of NO_3_ in 2000 estimated by the EMEP model (Simpson et al., 2012), _min: mineral layer chemistry, _org: organic layer chemistry (see Appendix S5 for details), _Tr: tree species (QurM: Mediterranean oak; Qurp: temperate oak, Fags: *Fagus sylvatica*, Pins: *Pinus sylvestris* + *P. nigra*, Pice: Picea abies, conf: “other” coniferous, deci: “other” deciduous), _Cli: climate zones (SubAtl: subatlantic, AtlN: Atlantic North, AtlS: Atlantic South, Bor: Boreal). Number of plots: 477, number of species: 170. Explanation of abbreviated species names: Acer cam: *Acer campestre*, Acer pla: *Acer platanoides*, Alchevul: *Alchemilla vulgaris*, Alliapet: *Alliaria petiolata*, Anemonem: *Anemone nemorosa*, Arenamon: *Arenaria montana*, Aruncdio: *Aruncus dioicus*, Asarueur: *Asarum europaeum*, Astragly: *Astragalus glycyphyllos*, Athyrfil: *Athyrium filix‐femina*, Cardaama: *Cardamine amara*, Cardabul: *Cardamine bulbifera*, Cardafle: *Cardamine flexuosa*, Cardahep: *Cardamine heptaphylla*, Cardaimp: *Cardamine impatiens*, Cardapen: *Cardamine pentaphyllos*, Carpibet: *Carpinus betulus*, Castasat: *Castanea sativa*, Chrysalt: *Chrysosplenium alternifolium*, Clemavit: *Clematis vitalba*, Corylave: *Corylus avellana*, Cratalae: *Crataegus laevigata*, Cratamon: *Crataegus monogyna*, Dryopaff: *Dryopteris affinis*, Dryopcar: *Dryopteris carthusiana*, Dryopdil: *Dryopteris dilatata*, Dryopfil: *Dryopteris filix‐mas*, Equisarv: *Equisetum arvense*, Equispra: *Equisetum pratense*, Equissyl: *Equisetum sylvaticum*, Fallocon: *Fallopia convolvulus*, Fragaves: *Fragaria vesca*, Geranrob: *Geranium robertianum*, Geum urb: *Geum urbanum*, Hellefoe: *Helleborus foetidus*, Hepatnob: *Hepatica nobilis*, Ilex aqu: *Ilex aquifolium*, Impatnol: *Impatiens noli‐tangere*, Impatpar: *Impatiens parviflora*, Lathymon: *Lathyrus montanus*, Lathypra: *Lathyrus pratensis*, Lathyven: *Lathyrus venetus*, Lathyver: *Lathyrus vernus*, Malussyl: *Malus sylvestris*, Mercuper: *Mercurialis perennis*, Mespiger: *Mespilus germanica*, Oreoplim: *Oreopteris limbosperma*, Oxaliace: *Oxalis acetosella*, Phegocon: *Phegopteris connectilis*, Polypvul: *Polypodium vulgare*, Potenste: *Potentilla sterilis*, Prunuavi: *Prunus avium*, Prunupad: *Prunus padus*, Prunuspi: *Prunus spinosa*, Pteriaqu: *Pteridium aquilinum*, Pyruspyr: *Pyrus pyraster*, Querccer: *Quercus cerris*, Quercpyr: *Quercus pyrenaica*, Quercrub: *Quercus rubra*, Ranunaur: *Ranunculus auricomus*, Ranunfic: *Ranunculus ficaria*, Ribesalp: *Ribes alpinum*, Ribesspi: *Ribes spicatum*, Rosa arv: *Rosa arvensis*, Rosa can: *Rosa canina*, Rosa pen: *Rosa pendulina*, Rubuscae: *Rubus caesius*, Rubusfru: *Rubus bifrons*, Rubusida: *Rubus idaeus*, Rumexact: *Rumex acetosella*, Rumexals: *Rumex alpestris*, Rumexsan: *Rumex sanguineus*, Silendio: *Silene dioica*, Sorbuari: *Sorbus aria*, Sorbuauc: *Sorbus aucuparia*, Sorbudom: *Sorbus domestica*, Sorbutor: *Sorbus torminalis*, Stellhol: *Stellaria holostea*, Stellnem: *Stellaria nemorum*, Ulmusgla: *Ulmus glabra*, Urticdio: *Urtica dioica*, Viciacra: *Vicia cracca*, Viciasep: *Vicia sepium*

Figure 2 is the biplot of the model of Table 1. There are two main gradients. One is determined by soil chemistry and runs from acid and nutrient‐poor (upper left) to neutral and nutrient‐rich (lower right). This gradient coincides with a gradient in species richness, which is low in the upper left and high in the lower right of the diagram. The species in the lower right are the ones that are characteristic for nutrient‐rich forest, for example, *Hepatica nobilis*,* Asarum europaeum*,* Impatiens noli‐tangere*. A second gradient is determined by tree species, mainly the contrast coniferous (lower left) versus deciduous (upper right). On the species side, this gradient runs from a dominance of ferns (e.g., *Dryopteris* spp., *Oreopteris limbosperma*) to a dominance of phanerogamic herbs (e.g., *Stellaria holostea*,* Alliaria petiolata*) and shrubs (e.g., *Crataegus monogyna*,* Prunus spinosa*). These ecological gradients are at an angle of c. 45° with the CCA axes (which is usual if two gradients are more or less equally important). The third and fourth axis (not shown) mainly separate the Mediterranean plots from all others, and are characterized by Mediterranean oak on the environmental side, and mediterranean species such as *Prunus mahaleb*,* Arenaria montana*, and *Lathyrus venetus* on the species side.

There is no single axis that clearly represents the effect of N deposition; this is also apparent from the canonical coefficients and their *t*‐values (not shown). Therefore, an extra analysis was run where the effect of N deposition was forced through the first axis. This is achieved by declaring N deposition as the only environmental variable and moving all other variables with a significant effect in Table 1 to the covariables. The resulting biplot (not shown) is difficult to interpret, although among the species that are best explained by the first axis (and have a positive correlation with N deposition) are clearly nitrophytic ones such as (Ellenberg *N* in brackets) *Moehringia trinervia* (7), *Geranium robertianum* (7), *Ranunculus repens* (7), and *Urtica dioica* (9). Also, the sample scores on this axis are nearly significantly correlated with the Ellenberg *N* scores (*R* = .10, *n* = 323, *p* = .08). We consider this as an indication that the effect of NO_3_ deposition in the model of Table 1 is not a spurious effect but most probably represents a real ecological effect.

#### 3.1.2 Multiple regression analysis of impacts of environmental variables on Ellenberg indicators and species numbers

Table 2 shows the effect of the environmental variables of the model given in Table 1, taken together per compartment, on the Ellenberg indicator scores and the number of species. This analysis mainly confirms the results of the CCA analysis, with soil chemistry and tree species as the most important explanatory variables for most indicators. The effect of soil chemistry is strongest for the indicator score for acidity, and the effect of tree species is strongest for the light score. The effect of climate is rather weak but strongest for the continentality score which is not unexpected. Two points deserve special attention: (1) the only indicator that is significantly influenced by N deposition is the nutrient indicator (*N*), which is again an indication for a real effect of deposition, and (2) the strong effect of the country on the number of species, which is an indication for an observer effect. This effect is still highly significant (*p* < 0.001, percentage variance uniquely due to countries: 10.0%) after accounting for differences in size of the plots (data not shown).

**Table 2:** Multiple regression of Ellenberg scores and number of species for the last relevé per plot using the model of Table 1

	Light (*L*)	Temperature (*T*)	Continentality (*K*)	Humidity (*F*)	Acidity (*R*)	Nutrients (*N*)	Number of species
Full model	53.5***	66.4***	54.4***	30.7***	67.8***	44.9***	44.5***
Country	2.3**	0.5 ns	1.5*	2.1 ns	1.9*	0.1 ns	10.5***
Soil chemistry	6.6***	1.1*	6.1***	4***	14.6***	5.9***	15.6***
Climate	0.3 ns	1.7**	2.3***	1.2 ns	1.8**	0.7 ns	1.5**
Tree species	16***	5***	2.7***	2.6*	2.1***	5.3***	1.7**
Deposition	0 ns	0.2 ns	0 ns	0 ns	0.1 ns	1.3**	0.2 ns
*N*	462	335	419	343	327	325	477

The first row gives the percentage variance explained by the full model and its overall significance, the other rows give the percentages variance uniquely attributable to the variables in each compartment, that is, the loss of explained variance on excluding these variables from the full model, and the significance of the corresponding change determined on the basis of *F*‐values. *N* = number of observations; unequal numbers are due to different numbers of relevés with too few species to calculate its Ellenberg score.

Significance levels: ***p < .001; **p < .01; *p < .05; ns, *p* > .05.

### 3.2 Relation between temporal change in vegetation and environment

#### 3.2.1 Multivariate analysis of impacts of environmental variables on the temporal change in species abundances

Table 3 gives the mean change in cover percentage per species for those species where this change was at least weakly significant (*p* < .1). This appears to be the case for only 13 of 297 species (note that at this number of species and *p* < .1 one would expect a false significance for 30 species). Apparently, the changes have been small for the individual species. There is no apparent pattern in the ecology of the species that most strongly changed. The three species that declined most strongly (*Rosa pendulina*,* Ranunculus platanifolius*, and *Ribes alpinum*), and the species that increased one‐but‐most strongly (*Athyrium distentifolium*) are typical mountain species. The strongest changes might also have a methodological background (*R. platanifolius* and *A. distentifolium* by being confused with *Ranunculus aconitifolius* and *Athyrium filix‐femina*, respectively, and *Anemone nemorosa* because of differences in observation date).

**Table 3:** Change in species cover between the first and last relevé

Species	*N*	Diff	*T*	*p*
*Rosa pendulina*	8	−1.17	−2.47	.043
*Ranunculus platanifolius*	3	−0.83	−5.00	.038
*Ribes alpinum*	12	−0.72	−1.91	.083
*Prunus avium*	54	−0.57	−1.99	.052
*Crataegus monogyna*	11	−0.54	−2.21	.052
*Potentilla erecta*	20	−0.52	−2.21	.040
*Lathyrus montanus*	13	−0.46	−3.25	.007
*Gymnocarpium dryopteris*	13	−0.43	−2.11	.056
*Hippocrepis comosa*	3	−0.41	−8.66	.013
*Dryopteris carthusiana*	74	−0.37	−1.71	.091
*Polypodium vulgare*	8	−0.21	−2.34	.052
*Athyrium distentifolium*	5	0.28	3.33	.029
*Anemone nemorosa*	47	6.75	2.82	.007

Only relevés made at intervals of at least 7 years were used. *N* = number of occurrences (i.e., number of plots with this species in one or both years), Diff = MEAN ([%cover in last relevé]—[%cover in first relevé]), *T* = *t*‐value of difference, *p* = *p*‐value of difference. The species given are those for which *N* > 2 and *p* < .1, in the order of increasing values for Diff.

**Figure 3:**
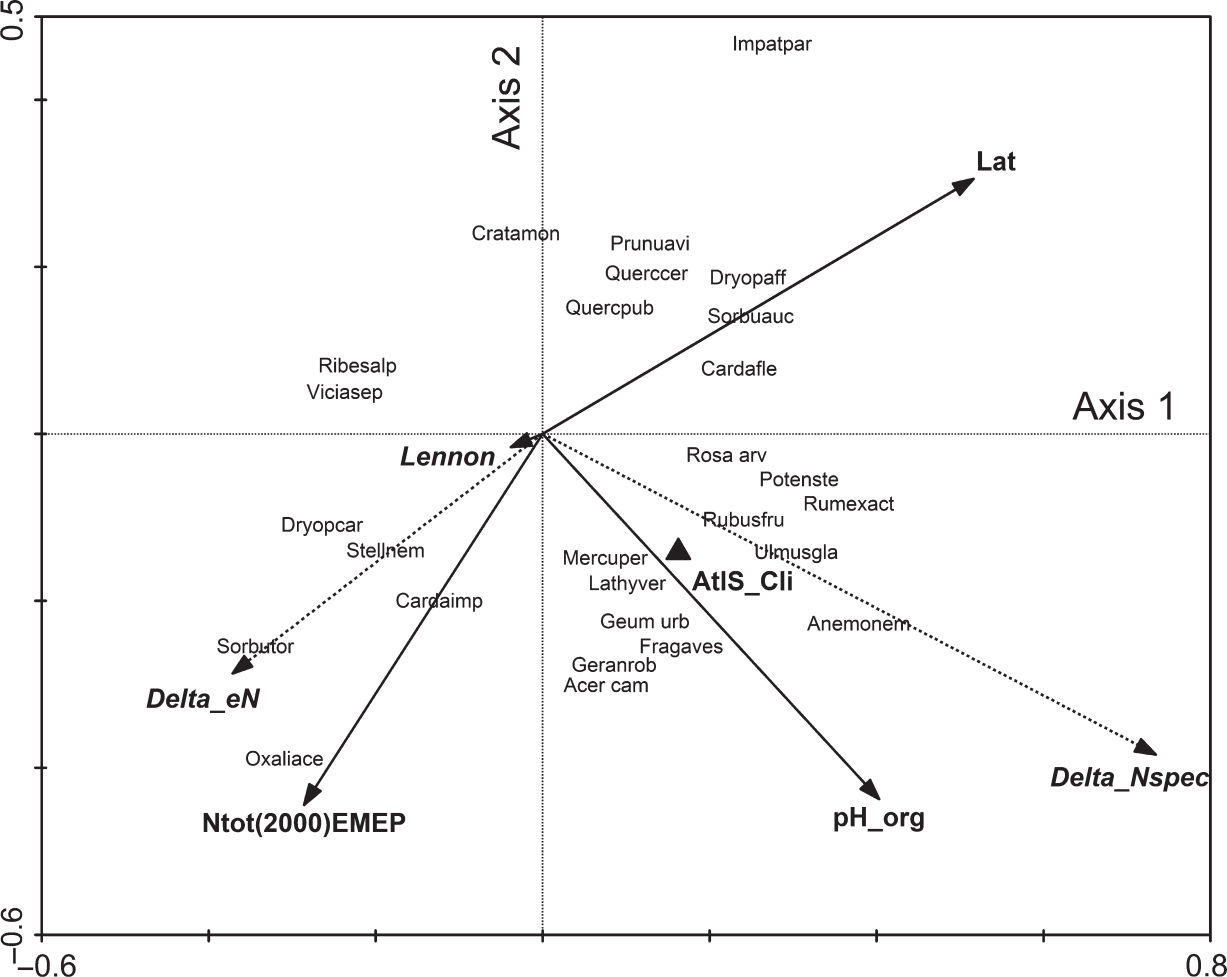
Biplot of the change model resulting from the RDA analysis of the difference (last relevé minus first relevé): first and second axis. See Table 4 for details of the model, percentage variance in the fitted values explained by this figure: 87%. Drawn arrows represent explanatory variables (see Figure 1 for explanation of their names), dotted arrows indicate the correlation of “passive” variables (that do not affect the ordination itself) with the axes (with Delta_eN: increase in Ellenberg *N* score per plot, Delta_Nspec: increase in number of species per plot, Lennon: dissimilarity between first and last relevé per plot). The plotted species are a selection of species with the highest percentage variance explained by the model. Number of plots: 99, number of species: 110, further explanation (incl. species codes) as in Figure 1

Table 4 gives the result of the RDA analysis of the effects of environmental variables on the temporal change in the vegetation. Figure 3 is the biplot of the model of Table 4. There is a significant effect of the total N deposition on the vegetation change, but again the biplot does not yield a clear ecological picture of what happened in the plots, although some nitrophytic species have a positive correlation with the N deposition (e.g., *Stellaria nemorum* [Ellenberg *N* = 7] or *Cardamine impatiens* [Ellenberg *N* = 8]). However, the direction in which the Ellenberg *N* score per plots increases nearly coincides with the direction in which N deposition increases (Figure 3). Again this is considered as an indication for a real effect of total N deposition on the vegetation.

**Table 4:** Forward selection of environmental variables in RDA to explain the variation of the change in vegetation per plot

Variable	Compartment	*F*	*p*	Percentage explained variance
Subatlantic	Climate	3.29	.003	3.0
Latitude	Climate	2.5	.050	3.0
N‐total (2000)	Deposition	3.57	.019	3.0
pH	Organic	2.55	.026	3.0
Atlantic North	Climate	1.58	.118	1
Temperate oak	Tree	1.68	.078	2
Further terms not given				
Sum if *p* < .05				12.0

Change is determined as the difference in abundance in the last relevé minus the first relevé of each species in each plot where the time lag between the first and last relevé is at least 7 years. Variable selection procedure as in Table 1, but no covariables were used. Eigenvalues: λ_1_ = 0.058, λ_2_ = 0.043, λ_3_ = 0.011, λ_4_ = 0.004, sum of eigenvalues standardized to unity, number of plots = 99, number of species = 110. Further explanation, see Table 1.

#### 3.2.2 Multiple regression analysis of impacts of environmental variables on changes in Ellenberg indicators and species numbers

Table 5 gives the change in Ellenberg scores and their significance. Of these, only the one for nutrient availability (*N*) significantly changed (increase, *p* = .01). Besides, the number of species per relevé highly significantly increased by 1.4 species (*p* < .001). Also, the Lennon dissimilarity between the two relevés of each plot is highly significantly different from zero, which indicates a considerable temporal species turnover.

**Table 5:** Change in Ellenberg indicator scores, number of species, and square‐root transformed Lennon dissimilarity index between the first and last relevé

Indicator	Mean	*N*	Change	*T*	*p*
Light (*L*)	5.0	152	0.016	0.489	.63
Temperature (*T*)	5.3	113	0.000	0.002	1.00
Continentality (*K*)	3.4	141	−0.023	−0.972	.33
Humidity (*F*)	5.2	128	0.046	1.648	.10
Acidity (*R*)	5.5	112	−0.013	−0.287	.77
Nutrients (*N*)	5.2	122	0.107	2.569	.01
Number of species	11.8	161	1.410	4.742	.00
SQRT (Lennon)	–	161	0.310	16.760	.00

Only relevés made at intervals of at least 7 years were used. Mean = mean value over both observation dates, *N* = number of observations (i.e., number of plots with Ellenberg value present in both years), Change = MEAN ([value in last relevé]—[value in first relevé]), *T* = *t*‐value of difference, *p* = *p*‐value of difference.

Table 6 gives the relation between temporal change in the Ellenberg *N* score and environmental variables. There is a significant effect of NO_3_ deposition, which influences the change in Ellenberg *N* in the expected direction, that is, an increase. The large negative value for the “undetermined” fit may be due to interaction, but there appeared to be no single interaction term that could significantly improve the fit of the model.

**Table 6:** Regression model to explain the change in Ellenberg *N* score

Variable	Compartment	Regression coefficient	Significance	TMV%
Latitude	Climate	−0.055	**	7.8
Fagus	Tree	−0.263	*	4.2
Mediterr. oak	Tree	−0.633	**	6.0
NO3 (1995)	Deposition	6.03E‐04	**	8.6
Undetermined				−14.5
Total expl. var.				12.1

The model is derived by stepwise exclusion of terms from a full model containing all variables included in Appendix S8, until only terms with a significant (*p *< .05) effect remain. TMV = top marginal variance, that is, the drop in explained variance when omitting this term from the model. Significance levels as in Table 2. The negative “unexplained” variance is due to interaction effects, and further explanation see text (*N* = 99).

One may get an idea of the effect of N deposition on the floristic change by multiplying the regression coefficient for NO_3_ deposition in Table 6 by the range of the deposition. The difference between the lowest and highest deposition (which are reached in Finland and the Netherlands, respectively) corresponds to a difference of c. one Ellenberg *N* unit. Assuming no effect at the lowest N deposition and taking account of an overall mean Ellenberg *N* value of 5.2 (Table 5), this might mean an increase of Ellenberg *N* value of c. 5 to c. 6 at the highest deposition and a mean value of all other variables. In floristic terms, this would mean a shift in conditions optimal for species such as *Holcus lanatus*,* Ilex aquifolium*,* Milium effusum*, or *Polygonatum multiflorum* to conditions optimal for, for example, *Calamagrostis epigeios*,* Digitalis purpurea*,* Galeopsis* spp., or *I. noli‐tangere*. There appeared to be no significant effect of deposition on the change in the number of species, nor on the Lennon dissimilarity; these variables were only significantly influenced by soil chemistry (data not shown).

## 4 Discussion and Conclusions

Using cover percentages as response variables and applying multivariate statistics, our study showed a significant relation between N deposition and change in the composition of ground vegetation in forests over Europe. In line with Van Dobben and De Vries (2010), it also yields a small but significant effect of NO_3_ deposition in a single point‐in‐time analysis. Although the effect of NO_3_ deposition on individual species cannot be clearly identified, the effect on the vegetation as a whole is a shift toward nitrophytic species. It is very hard to determine whether the change in the vegetation coincides with a change in the NO_3_ deposition itself because the period over which the change was considered is different per plot both in starting point and in length. Between 1995 and 2000, the deposition inferred by the EMEP model significantly decreased (*p* << .001) for both N‐total, NH_4_, NO_3,_ and SO_4_. The vegetation in the last relevé per plot is best explained by the deposition of NO_3_ in 2000, while the change is better explained by the deposition of N‐total in 1995 (but note that a better fit for a certain date is caused by differences in the spatial pattern at the two dates and not by the absolute amounts of deposition).

Our results can probably also be explained on the basis of Bernhardt‐Römermann et al.'s (2015) hypothesis that vegetation change is primarily driven by the accumulated deposition before the start of the observations. Although we did not explicitly test this, it is highly plausible that the accumulated deposition at the start is strongly correlated to the deposition during the observation period considering that spatial patterns of N deposition have been rather stable in the last 30–40 years.

In the RDA analysis of the relation between vegetation changes and environment, all plant species were included. However, in our CCA analyses of the relation between vegetation and environment at a single point in time, we had to exclude rare plant species as very heterogeneous data sets cannot be analyzed by CCA related techniques. According to the “random‐loss” hypothesis (Suding et al., 2005), the probability of extinction due to nitrogen deposition is higher for rare species than for common species. If this effect is important in our case, there would be a negative relation between N deposition and species number. However, which we did not find such a relation (Table 2), and therefore, we conclude that the effect of N deposition comes about through changes in the abundance of common species rather than through loss of rare species, which agrees with De Schrijver et al. (2011). In this respect, the response of forest understorey vegetation to N deposition seems to be different from the response of grassland, where loss of rare species was shown in several studies (Maskell et al., 2010; Payne et al., 2013; Stevens et al., 2004, 2010).

It is difficult to indicate the exact nature of the vegetation change induced by N deposition. The most straightforward indication for an effect of N deposition on the vegetation would be a significant temporal change in the abundance of indicative species, but to our knowledge, such an indication was not found in any study over a large geographical extent incl. the present one. A change is only apparent for generalized measures viz. those derived from multivariate statistics or indicator values. Apparently, there were no large changes in single species, but rather small changes occurring over a wide range of species. This is probably due to the very large spatial species turnover at the geographical extent of ICP‐Forests. In the total set of 776 plots with vegetation data, 546 species were observed but of these only 49 occur in more than 5% of the plots. Therefore, even if there is a general increase of nitrophytic species, these are regionally different species and consequently the number of observations per species is too low to detect a significant change. Not surprisingly, the species with the strongest correlation with N deposition in the multivariate analysis are mostly very common ones (*M. trinervia*, frequency = 20%; *G. robertianum*, 10%; *R. repens*, 3%; *U. dioica*, 12%). Methods based on generalized measures per species (i.e., derived from multivariate statistics or indicator values) are not hampered by the large spatial turnover and are therefore far more sensitive to effects of environmental variables.

Despite their obvious shortcomings (Wamelink, Goedhart, Van Dobben, & Berendse, 2005), we consider Ellenberg's indicators for nutrients (*N*) and acidity (*R*) as reliable estimators for the response of forest understorey vegetation to soil nitrogen and acidity status. Van Dobben et al. (1999), in a long‐term experimental study in ground vegetation, found a strong relation between mean Ellenberg *N* and Ellenberg‐*R* scores per relevé on the one hand and experimentally added nitrogen or lime, respectively, on the other hand. Also, Diekmann and Falkengren‐Grerup (1998), in an observational study over 661 forest sites, showed that Ellenberg *N* is a reliable estimator for the measured N‐mineralization rate.

Our results are at variance with those of Verheyen et al. (2012), who did not find a significant effect of N deposition on long‐term changes in forest understorey vegetation. Several reasons may be found to explain this difference, the most obvious being that Verheyen et al. used a much longer time interval (c. 20–60 years), limiting their study to forest reserves, as compared to the relatively short time interval (7–11 years) and a focus on production forests in our study. Therefore, changes in light climate and litter quality (which are in turn caused by long‐term changes in the tree layer) are much larger in that study compared to ours, and Verheyen et al. (2012) concluded that most of the long‐term changes in vegetation are due to these factors. In contrast, we did not find any effect of light climate (based on the change in Ellenberg's light score, cf. Table 5). Another difference is that Verheyen et al. (2012) only used total N deposition as an explanatory variable, while we included both NO_3_, NH_4,_ and total N (i.e., the sum of NO_3_ and NH_4_) as potential explanatory variables.

Our results are generally in line with Dirnböck et al. (2014) who studied temporal trends in vascular plant species cover and diversity, based on monitoring data during a comparable period (a 10–15 year period between 1994 and 2011) in 28 forest stands in a north–south gradient through Europe (their sites partly coincide with our sites). They found a temporal trend that can be summarized as a gradual replacement of species with a low Ellenberg *N* value by species with a high Ellenberg *N* value, at a rate that increases with increasing N deposition (expressed as critical load exceedance). These authors did not find a significant overall trend in species number; both sites with a significant increase, a significant decrease, and no significant change occurred. In contrast, we found a strong and highly significant increase in the number of species per plot, for which we do not have an apparent explanation. Possible explanations include (1) N deposition, (2) climate change, and (3) observer effects.

Hülber et al. (2008), in a resampling study of 14 intensively monitored forest plot in a 90 ha region, found a considerable observer effect (quantified as the distance in ordination space between synchronous observations of the same plots by independent teams), but these authors argued that the distance between subsequent observations of a single plot (with a time interval of 12 years) is significantly larger than the mean observer effect. An increase in the number of species is often found in re‐evaluations of permanent plots (e.g., Grabherr, Gottfried, & Pauli, 1994; Thimonier, Dupouey, Bost, & Becker, 1994), and this increase may be partly an observer effect. Archaux et al. (2009) studied observer bias in the French ICP‐Forests plots and reported c. 20% of the species in the ground vegetation of each 50 × 50 m plot to be overlooked and c. 5% to be misidentified. However, their data include the moss layer so in our analysis these percentages might be somewhat lower. An important factor determining the overlooking rate was the familiarity of the observers with the local vegetation. Although re‐evaluation of a given plot is not necessarily undertaken by the same observer, we expect that on average the observer's familiarity with the plot's vegetation increases over time. Also, ICP‐Forests’ manual (Canullo et al., 2011) does not forbid to take the species list of previous observations into the field, which may also decrease the overlooking rate in subsequent observations. Interestingly, Verheyen et al. (2012) reported a considerable temporal species turnover but no significant increase in species number over a time interval that is so large that subsequent observations were most probably made by different persons.

We found a c. 10% increase in species number (Table 5) which means that the overlooking rate in the last relevé would be c. half of the overlooking rate in the first relevé if it is the only explanation for the increase in species number (note that misidentification does not influence the species number). This decrease in overlooking rate seems very strong, also in light of the rather constant overlooking rate of c. 20% between various studies (see Archaux et al., 2009: Table 3 and Allegrini, Canullo, & Campetella, 2009). Therefore, there might also be a real increase in species number, the cause of which is unknown. N deposition is improbable as a cause because there appeared to be no significant relation between species number and deposition at a single point in time (Table 2), and neither was there a significant relation between N deposition and the increase in species number. In this respect, our results confirm those of De Schrijver et al. (2011), who in a meta‐analysis of N addition experiments, noted a loss of species after N addition in grassland and heathland, but no clear effect on species number in forest. Also climate change is rather improbable as a cause as the Ellenberg scores for both temperature and continentality hardly showed any change over time (Table 5). Vice versa, it might be argued that the increase in Ellenberg *N* score is an observer effect. However, we cannot find any reason why observers would be less prone to overlook nitrophytic species in subsequent re‐evaluations of the plots.

Earlier studies based on (subsets of) ICP‐Forests or other data either did not find a relation between N deposition and vegetation change (e.g., Campetella, Canullo, & Allegrini, 2006; Seidling, 2005; Verheyen et al., 2012), or, in single point‐in‐time analyses, only a small although significant effect (e.g., Seidling & Fischer, 2008; Seidling et al., 2008; Van Dobben & De Vries, 2010). The present study agrees with the previous single point‐in‐time analyses, but we found a smaller effect for NH_4_ or N‐total than for NO_3_. This is surprising as experimental results in short vegetation (grassland or heathland) suggest a stronger effect of NH_4_ than NO_3_ (De Graaf, Bobbink, Roelofs, & Verbeek, 1998; Kleijn, Bekker, Bobbink, de Graaf, & Roelofs, 2008; Paulissen, van der Ven, Dees, & Bobbink, 2004; Van den Berg, Peters, Ashmore, & Roelofs, 2008). High concentrations of NH_4_ in soil solution or surface water may even be toxic to sensitive species (Bobbink et al., 2003). However, these effects are less clear in forest. In a culture experiment involving a large number of common understorey species of deciduous forests in southern Sweden, Falkengren‐Grerup (1995b) showed that acid‐tolerant species grow equally well in NH_4_ alone as in a mixture of NH_4_ and NO_3_, while species of less acid soils prefer a mixture but also grow on NH_4_ alone. These results were confirmed for a smaller number of species in field observations by Olsson and Falkengren‐Grerup (2000). Therefore, the effects of nitrogen deposition in forest may be different from those in short vegetation, both for the total number of species and for the difference between NO_3_ and NH_4_. On the other hand, we cannot rule out the possibility that the stronger effect of NO_3_ compared to NH_4_ is an artifact caused by the larger uncertainty in NH_4_ deposition estimated by EMEP or by the strong spatial variability in NH_4_ deposition, which does not become apparent at EMEP's spatial scale of 50 km.

At present, the effect of deposition on understorey vegetation is limited (cf. Van Dobben & De Vries, 2010). The significant effect of deposition on the floristic change in spite of a decreasing trend in deposition indicates that there is a considerable lag in the response of the vegetation to deposition. Bernhardt‐Römermann et al. (2015) found indications that the effect of N deposition only becomes visible when growth‐limiting factors other than N (e.g., light) are in sufficient supply. Jonard et al. (2015), in a study of tree nutritional status in ICP‐Forests plots, found that increased tree growth (probably due to elevated CO_2_ and N availability) and resulting higher nutrients demands, led to decreasing foliar concentrations, down to levels that are characterized as “low” or “deficient” for P (and sometimes also other nutrients, such as S and Mg) in a number of tree species. If this is also the case for understorey vegetation, growth might be progressively more limited by a low supply of, for example, P, which might mask the effect of N deposition. However, the present data do not allow an estimation of the magnitude of such an effect, and therefore, a prediction of future developments is not possible either. When other environmental factors change, for example, due to climate effects, the effects of N deposition may become more prominent over time, which can only be detected if the present or a comparable form of intensive monitoring is continued (Fischer et al., 2011).

## Conflict of Interest

None declared.
